# Book Reviews

**Published:** 1897-04

**Authors:** 


DISEASES OF THE STOMACH, A Text-Book for Practitioners and Students. By MAX EINHORN, M. D., Instructor in Clinical Medicine at the New York Post-Graduate Medical School and Hospital; Visiting Physician to the German Dispensary. Pp. 496. New York: William Wood & Co. 1896.

A few years ago there was very little literature relating to the subject of which this volume treats outside of a few short paragraphs or brief chapters found in general treatises on the practice of medicine. Now, however, the field is becoming rich in this regard and with the possible danger of its being overworked.

It cannot be denied, however, that there is always room for good literature upon any branch of medicine, and we welcome this book as one of the best of its kind. The author speaks in his preface of some contributions to the advancement of our knowledge on this branch of medicine that have been made by Drs. Charles G. Stockton and Allen A. Jones, of Buffalo—names that are familiar to our readers for their special work in this department of medicine.

In the first chapter the author treats of the anatomy and physiology of the stomach, and in the second passes directly to the consideration and methods of examination. In this, minute directions are given with reference to physical examination, sounds of the stomach, gastroscopy, gastrodiaphany, or transillumination of the stomach, the Röntgen rays and examinations of the functions of the stomach. In this latter all known methods are considered and given in considerable detail.

The third chapter takes up diet, and discusses animal, vegetable and liquid foods, the utilisation of foods, diet in health, and diet in acute and chronic diseases of the stomach. There is a field here for much instruction, and the author has improved his opportunity. In this chapter he quotes occasionally from Gilman Thompson's recent work on practical dietetics, that has been noticed in these columns.

In Chapter IV. Einhorn takes up local treatment of the stomach, beginning with lavage, and advancing to the gastric douche and spray, and finally ending with electricity. He makes reference in this chapter to Dr. Stockton's new gastric electrode, and to his paper on Clinical results of gastric faradisation.

Organic diseases, with constant lesions, form the subject of Chapters V., VI., VII. and VIII., in which acute and chronic gastric catarrh, ulcer of the stomach, erosions of the stomach, and cancer of the stomach are considered. It is a melancholy fact that the latter disease is constantly on the increase. It has been shown by Dr. Joseph D. Bryant, of New York, that the death-rate from cancer in that city during the last ten years is 2 1-17 per cent. of the total mortality, while during the preceding ten years it was only 1.82 per cent. Sadly enough it is a malady that seems to be beyond the domain of cure. Einhorn goes into the subject very fully, and presents whatever is known of scientific value at the present day.

Chapters IX., X. and XI. deal with functional diseases with variable lesions, such as grow out of hypersecretion. Ischochymia, an affection characterised by the constant presence of food in the stomach, is very interestingly considered in Chapter XI. Next to the subject of hyperchlorhydria, this is perhaps the most interesting functional derangement of the gastric economy that is met with in practice, and some of the observations on the subject are quite new.

Abnormal conditions with reference to the size, shape, and position of the stomach are taken up in Chapter XII.; nervous affections of the stomach are considered in Chapter XIII., necessarily a long chapter; and the condition of the stomach in diseases of other organs furnishes the topic for the concluding brief chapter.

The book is well indexed, and is also handsomely printed and bound. We shall find ourselves seriously mistaken if this treatise does not become a work of study and frequent reference by all practitioners and teachers of medicine. It is a valuable contribution to the literature of the subject, and stands in marked contrast to many of the works that are foisted on an unwilling profession by eager authors and zealous publishers.

---

ANOMALIES AND CURIOSITIES OF MEDICINE. Being an encyclopedic collection of rare and extraordinary cases, and of the most striking instances of abnormality in all branches of medicine and surgery, derived from an exhaustive research of medical literature from its origin to the present day, abstracted, classified, annotated and indexed. By GEORGE M. GOULD, A. M., M. D., and WALTER L. PYLE, A. M., M. D. Imperial 8vo, 968 pages, with 295 illustrations in the text and twelve half-tone and colored plates. Philadelphia: W. B. Saunders, 925 Walnut street. 1897.

The curious in literature, as the author of this work very justly remarks, always has a fascination, and we are sure that though the work in the preparation of this book has been enormous, yet it must have been a genuine pleasure for its compilers. It is not a book that comes within the domain of the reviewer in the ordinary sense; yet, nevertheless, it is one that merits more than ordinary notice.

Heretofore a writer has been obliged to delve through libraries, bookshelves and tomes, in order to obtain information with reference to a given anomaly or "freak" that may be germain to his subject. Now, however, he will be enabled to find these either published in detail or with complete references, in many instances illustrated, within this single volume, and with which the thoughtful men will proceed to ornament his library at once.

There are eighteen chapters with the following titles: Chapter I., Genetic anomalies; II., Prenatal anomalies; III., Obstetric anomalies; IV., Prolificity; V., Major terata; VI., Minor terata; VII., Anomalies of stature, size and development; VIII., Longevity; IX., Physiologic and functional anomalies; X., surgical anomalies of the head and neck; XI., Surgical anomalies of the extremities ; XII., Surgical anomalies of the thorax and abdomen ; XIII., Surgical anomalies of the genito-urinary system ; XIV., Miscellaneous surgical anomalies ; XV. Anomalous types and instances of disease ; XVI., Anomalous skin diseases : XVII., Anomalous nervous and mental diseases ; XVIII., Historic epidemics.

The value of this book in medico-legal cases will necessarily be appreciated by medical witnesses. Again, to the surgeon it will become a constant book of reference, for in it are recorded many surgical anomalies of the entire organism. Not the least curious are some of the recoveries that have followed multiple injuries, or those produced under unusual circumstances, causing great destruction of tissue. Many "freaks" are sketched in this work, and some excellent illustrations of several of those that are familiar to frequenters of the dime museums are here published.

A historical sketch of the noted Siamese twins is given in the book, and reference is made to the fact that the late Dr. William H. Pancost formed one of a committee of the Philadelphia College of Physicians that made a post mortem investigation of the case, which formed the subject of an interesting report to the society.

The obstetrician and gynecologist will be much interested in examining the portions of this book that bear relation especially to their branches of medical science. Some of the most remarkable anomalies, curiosities and freaks in the whole human economy are to be found recorded in the section of the book relating to these subdivisions of medicine.

Finally, it is well printed on good paper, although its size and weight demands a stronger binding than pertains to leather, and we should advise those who purchase the volume to secure the half-morocco covers. The work of the compilers has been most praiseworthy, and the profession of medicine ought to feel indebted to these tireless delvers into the literature of the centuries from which they have rescued and recorded so much of enduring interest. We predict a large sale for this remarkable volume.

